# Relationship between alterations of urinary microbiota and cultured negative lower urinary tract symptoms in female type 2 diabetes patients

**DOI:** 10.1186/s12894-019-0506-0

**Published:** 2019-08-22

**Authors:** Jiawei Chen, Jie Zhao, Ying Cao, Guihao Zhang, Yang Chen, Jialei Zhong, Weina Huang, Jiarong Zeng, Peng Wu

**Affiliations:** 1 Department of Urology, Nanfang Hospital, Southern Medical University, Guangzhou, 510515 China; 2 School of Pharmaceutical Sciences, Southern Medical University, Guangzhou, China; 3 Department of Endocrinology and Metabolism, Nanfang Hospital, Southern Medical University, Guangzhou, 510515 China

**Keywords:** Diabetes mellitus, Lower urinary tract symptoms, Urinary microbiota, Hemoglobin A1c

## Abstract

**Background:**

Lower urinary tract symptoms (LUTS) is the most common complication of diabetes. However, the underlying pathogenesis of cultured negative LUTS (cn-LUTS) in diabetic patients has not been well understood. Numerous evidence indicates that urinary dysbiosis is related to urologic disorders. We aim to study alterations of the urinary microbiota of cn-LUTS in type 2 diabetes (T2D) patients.

**Methods:**

Female T2D patients and controls were recruited and requested to finish the American Urological Association Symptom Index. Mid-stream urine was collected for culturing and extracting DNA. Microbial diversity and composition were analyzed by targeting to 16S rDNA. Linear discriminant analysis effect size (LEfSe) was carried out to identify significantly different bacteria.

**Results:**

32 female T2D patients and 26 controls were enrolled. No significant differences in alpha diversity were observed between patients and controls. However, statistically decreased richness (ACE index and Chao 1 index, 85.52(13.75, 204.84) vs. 129.82(63.89, 280.30) and 83.86(11.00, 210.77) vs. 125.19(62.00, 251.77), *P* = 0.005; Observed Species, 76(10, 175) vs. 98(54, 234), *P* = 0.011) and decreased species diversity (Shannon index, 1.37(0.04, 3.48) vs. 2.09(0.98, 3.43), *P* = 0.033; Simpson index, 0.46 (0.06, 0.99) vs. 0.23(0.07, 0.64), *P* = 0.029) were shown in moderate-to-severe LUTS group and high Hemoglobin A1c group, respectively. A significant difference of beta diversity was found between T2D patients and controls and T2D patients with different severity of cn-LUTS as well as the different level of Hemoglobin A1c. LEfSe revealed that 10 genera (e.g., *Escherichia-Shigella* and *Klebsiella*) were increased and 7 genera were decreasing in T2D patients, 3 genera (e.g., *Escherichia-Shigella* and *Campylobacter*) were increased and 16 genera (e.g., *Prevotella*) were reduced in moderate-to-severe LUTS group, 2 genera (*Escherichia-Shigella* and *Lactobacillus*) were over-represented and 10 genera (e.g., *Prevotella*) were under-represented in high Hemoglobin A1c group. Finally, Hemoglobin A1c was found positively correlated with the total score of the American Urological Association Symptom Index (*r* = 0.509, *P* = 0.003).

**Conclusions:**

Urinary dysbiosis may be related to cn-LUTS in female T2D patients. A better understanding of urinary microbiota in the development and progression of cn-LUTS in female T2D patients was necessary. The severity of cn-LUTS was correlated to hyperglycemia and chronic hyperglycemia might induce or promote cn-LUTS by influencing urinary microbiota.

**Electronic supplementary material:**

The online version of this article (10.1186/s12894-019-0506-0) contains supplementary material, which is available to authorized users.

## Background

Diabetes is burgeoning worldwide due to an increase in aging population and changes in eating habits [1]. Chronic hyperglycemia is associated with damage, dysfunction, and failure of multiple organ systems, including the genitourinary system. 80% of individuals with diabetes are accompanied by lower urinary tract symptoms (LUTS) [1, 2]. Although diabetes-induced LUTS is not life-threatening, it seriously affects patients’ life quality. Most diabetic patients are likely suffering from LUTS without evidence of urinary tract infections (UTI), which are refractory and hardly benefit from the conventional treatment. For these patients, it is supposed that the potential pathogenesis of LUTS may be linked to polyuria, oxidative stress and autonomic neuropathy induced by chronic hyperglycemia [3]. However, the underlying pathogenesis of cultured negative LUTS (cn-LUTS) induced by diabetes has not been fully appreciated.

Microbiota has been increasingly considered as an essential factor in human health and disease [4–8]. Traditionally, specimens from mid-stream urine were usually considered being sterile for not enough uropathogens were yielded under standard clinical cultivation procedures. However, the dogma of ‘sterile urine’ has been broken. Recent studies have revealed that urine samples from healthy individuals inhabit numerous microorganisms [9]. Moreover, alterations of urinary microbiota were found in various urologic disorders [10–13]. Decreasing diversity and richness of urinary microbiota were found in female overactive bladder patients in our previous study [10]. Pearce et al. found that, compared to healthy controls, the proportion of *Gardnerella* in urge urinary incontinence patients was increased while the proportion of *Lactobacillus* was decreasing [11]. Siddiqui et al. reported that the diversity of urinary microbiota in interstitial cystitis patients was lower than that in healthy individuals and the proportion of *Lactobacillus* was increased [12].

An increased level of urine glucose induced by chronic hyperglycemia changes the microenvironment of the urinary tract which might further change the urinary microbiota. Liu et al. reported that urine specimens collected from female Type 2 diabetes (T2D) patients had a decreasing diversity and richness urinary microbiota [14]. They also found that increased *Actinobacteria* phylum and decreasing *Akkermansia muciniphila* was associated with the level of fasting blood glucose levels [14]. Accordingly, we suggested that alterations of urinary microbiota might also take part in the occurrence of cn-LUTS in diabetic patients. The goal of our study was to characterize urinary microbiota in Chinese T2D females by comparing the alpha and beta diversity as well as the specific genus and to explore the potential pathogenesis of diabetes-induced cn-LUTS from the characteristics of urinary microbiota.

## Methods

### Patient enrollment and urinary samples collection

This study was subject to approval by the Ethical Committee of Southern Medical University. From June 2017 to December 2017, females who diagnosed with T2D and healthy female volunteers, age between 40 and 70, were approached for participation. Participants with medical conditions that could interfere with voiding function such as presence of UTI, known abnormal anatomical abnormalities of urinary tract, hereditary and congenital diseases (spina bifida, spinal meninges, etc.), neurological diseases (Guillain-Barré syndrome, multiple sclerosis, etc.), LUTS caused by drug abuse, history of spinal injury, history of hysterectomy, previous major pelvis surgery and evidence of vaginal prolapse were excluded. Patients during the menstrual period or with medical conditions which may interfere with urinary microbiota such as recent antibiotics usage or indwelling catheter (within one month) were also excluded. All participants were requested to offer demographic information and complete the AUA-SI to evaluate the severity of LUTS. Before evaluation, all participants were briefed on the procedure and asked to write informed consent. Mid-stream urine (50 ml) was collected with the labial separation which was supervised by the author (Weina Huang) and 20 ml was left for standard cultivation to exclude UTI. Within 1 h, the rest specimens were immediately shifted to the laboratory and centrifuged at 16,000 g for 10 min. Pellets were kept at − 80 °C until further processing.

### DNA extraction, PCR, and MiSeq sequencing

DNA extraction was carried out using the cultured cells protocol supplied with the DNeasy Blood and Tissue Kit (Qiagen, Germany) in a laminar flow hood to avert contamination. The concentration of extracted DNA was tested by the Nanodrop ND-1000 spectrophotometer (Thermo Electron Corporation, USA). Specific primer sets for V3-V4 regions were chosen to perform PCR amplification of 16S rDNA. To assess the contribution of extraneous DNA from reagents, extraction negative controls (no urine) and PCR negative controls (no template) were included as the blank controls. Qiaquick PCR purification kit (Qiagen, Valencia, CA) was selected for purifying the final PCR products from unincorporated nucleotides and primers. Purified samples were normalized to equal DNA concentration and sequenced by Illumina Miseq sequencer (Illumina, Inc., USA).

### Statistical analysis

Differences in the demographic and clinical characteristics between groups were tested by Student’s *t*-test or Mann-Whitney *U* test (for continuous variables) as well as Pearson’s chi-square test or Fisher’s exact test (for count data). Pearson’s correlation was implemented to detect the relations between clinical data and indices of bacterial bioinformatics. All data were analyzed using SPSS (version 22). All tests were two-sided and *P*-values < 0.05 was considered statistically significant. PASS programme (PASS 11, NCSS, Kaysville UT, USA) provides estimates of power by simulation.

### Bioinformatics analysis

Quantitative Insights Into Microbial Ecology (QIIME, version 1.80) was applied to create an operational taxonomic units (OTUs) table at a default similarity level of 97% based on the 16S rDNA sequence data [15, 16]. Subsequently, chimera detection was performed through the UCHIME method [17]. The representative sequence of each OTU was aligned to Silva and Greengenes database using Ribosomal Database Project Classifier [18].

Five alpha diversity was calculated by mothur software (version 1.31.2) indexes in QIIME including the Observed Species, Chao 1 index, ACE index, Shannon index, and Simpson index. The difference of alpha diversity was evaluated by Mann-Whitney *U* test in R (version 3.03). Observed Species, Chao1 index, and ACE index represented bacterial richness, while Shannon index and Simpson index were quantitative measures of bacterial diversity that reflecting both species richness and evenness.

To compare microbial composition between groups, principal coordinate analysis (PCoA) was applied on Bray Curtis, weighted UniFrac and unweighted UniFrac distance metrics to generate three-dimensional plots in QIIME. Multiple Response Permutation Procedure (MRPP, a nonparametric test which applied to test beta diversity values between groups) was performed to test the differences of Bray Curtis, weighted UniFrac, unweighted UniFrac distance metrics between groups in QIIME.

Significant bacteria on the relative abundance was tested by Metastats Test. Initial *p*-value was corrected with the method of Benjamini-Chochberg in R [19]. False discovery rate (FDR) represented the corrected *p*-value and FDR < 0.05 was considered significant. To identify significantly different bacteria as biomarkers between groups, taxa summaries were reformatted and inputted into Linear discriminant analysis effect size (LEfSe) via the Huttenhower Lab Galaxy Server [20]. Firstly, the Kruskal-Wallis rank sum test and Mann-Whitney *U* test were applied to identify the significantly different bacteria. Subsequently, linear discriminant analysis (LDA) was used to score the effect size of the different bacteria. Only taxa with logarithmic LDA score greater than 2 were considered significantly enriched.

## Results

### Demographic and clinical characteristics of participants

Sixty five female volunteers were enrolled, including 35 T2D patients and 30 healthy controls. All the urine samples from these participants were verified cultured negative during the standard culture procedure. However, 3 samples from patients and 4 from controls were excluded due to the sequencing reads could not be achieved. With 32 patients in diabetes group and 26 subjects in the control group (α = 0.05; β = 0.2), we would have 95% power to detect differences at the 0.05 significance level (alpha) using a two-sided Mann-Whitney *U* test and have 96% power to detect differences at the 0.05 significance level (alpha) using a two-sided Student’s *t*-test. Detailed comparisons between T2D patients and healthy controls were shown in Table 1. Except for fasting blood glucose, no significant differences were observed in the demographic and clinical characteristics between diabetes group and control group (for example, age, 56.969 ± 8.014 vs. 57.615 ± 9.239; body mass index, 23.739 ± 4.379 vs. 24.298 ± 3.120; hypertension rate, 14(43.8%) vs. 8(30.8%); etc.). Besides, higher scores of American Urological Association Symptom Index (AUA-SI) (total score, 9.219 ± 6.904 vs. 2.846 ± 3.319; storage score, 5.375 ± 4.612 vs. 1.846 ± 1.617; emptying score, 3.844 ± 4.573 vs. 1.000 ± 2.482) were found in T2D patients (*P* < 0.05).

**Table 1:** Comparisons of demographic and clinical characteristics as well as bacterial alpha diversity between diabetes group and control group

	Diabetes (*n* = 32)	Control (*n* = 26)	*P* value
Demographic Characteristics			
Age (y)	56.969 ± 8.014	57.615 ± 9.239	0.776
Body mass index (kg/m2)	23.739 ± 4.379	24.298 ± 3.120	0.587
Menstrual status [no.(%)]			0.600
Premenopausal	8 (25.0%)	5 (19.2%)	
Postmenopausal	24 (75.0%)	21 (80.8%)	
Reproductive status [no.(%)]			0.448
Fertile	32 (100%)	25 (96.2%)	
Sterile	0 (0%)	1 (3.8%)	
Clinical Characteristics			
Duration of diabetes (y)	6.906 ± 4.748	N/A	
Fasting blood glucose (mmol/L)	7.913 ± 3.264	5.182 ± 0.592	< 0.001
Hemoglobin A1c (%)	8.088 ± 2.168	N/A	
Retinopathy [no.(%)]	10 (31.3%)	N/A	
Peripheral neuropathy [no.(%)]	27 (84.4%)	N/A	
Hypertension [no.(%)]	14 (43.8%)	8 (30.8%)	0.311
Creatinine (μmol/L)	65.156 ± 52.802	58.423 ± 8.420	0.178
Estimated glomerular filtration rate (ml/min/1.73m^2^)	104.513 ± 38.578	94.576 ± 16.170	0.193
American Urological Association Index			
Total score	9.219 ± 6.904	2.846 ± 3.319	< 0.001
Storage score	5.375 ± 4.612	1.846 ± 1.617	0.001
Emptying score	3.844 ± 4.573	1.000 ± 2.482	0.001
Parameter of Bacterial Alpha Diversity			
Observed species	88 (11, 245)	135 (15, 258)	0.166
Chao1	103.60 (14.00, 272.00)	149.50 (29.00, 339.00)	0.107
ACE index	104.30 (16.50, 416.70)	146.10 (40.70, 428.60)	0.077
Shannon index	1.70 (0.04, 3.48)	1.78 (0.01, 3.54)	0.552
Simpson index	0.31 (0.06, 0.99)	0.38 (0.07, 1.00)	0.381

Data were presented as mean ± SD for continuous variables and n (%) for counts in demographic and clinical characteristics. Parameters of bacterial alpha diversity were reported as median (range). N/A, not applicable. ※No data were missing

### Comparisons of bioinformatics between T2D patients and controls

**Fig. 1:**
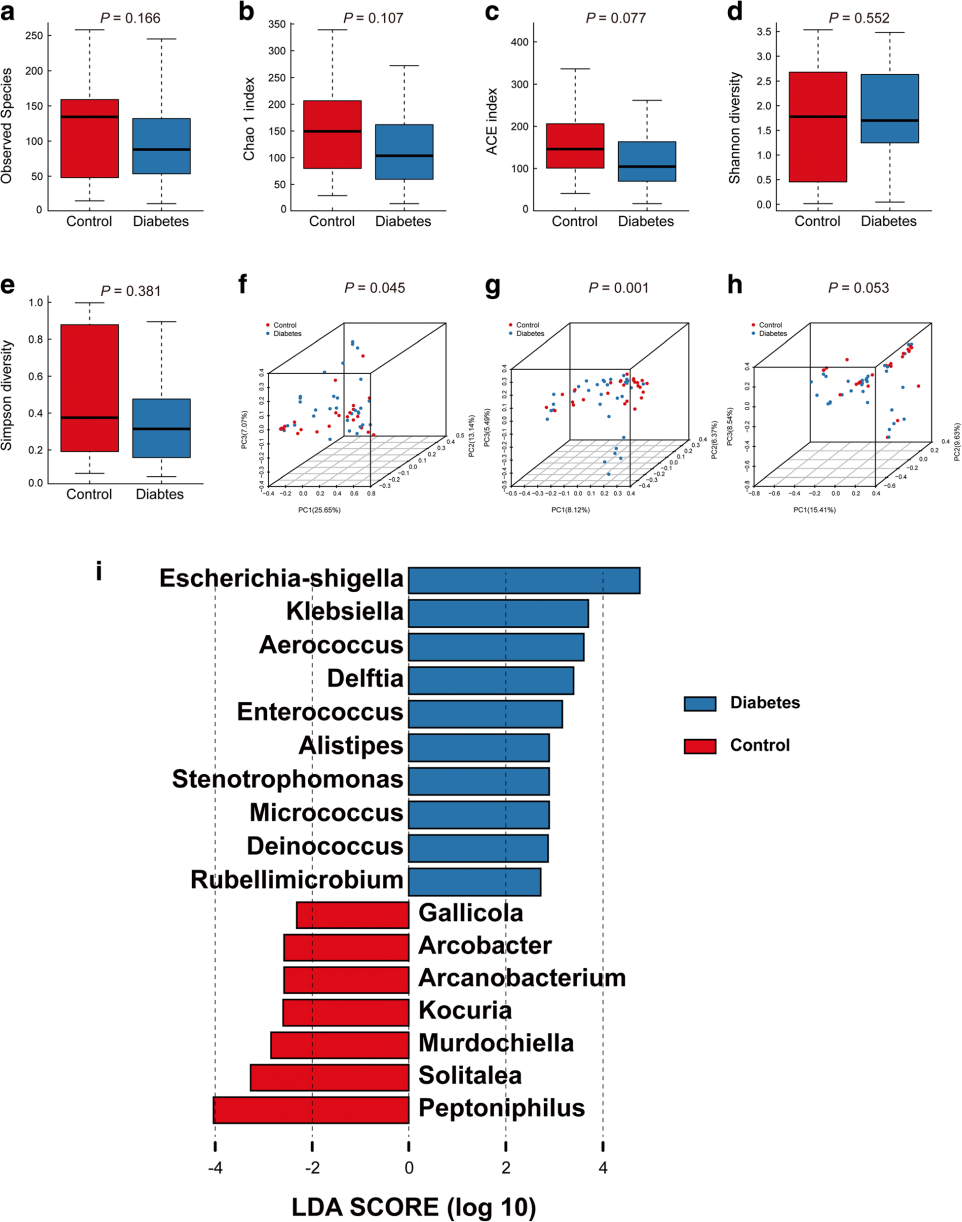
Alpha diversity and principal coordinate analysis for the control group and diabetes group urinary microbiota. Observed Species **(a)**; Chao1 index**(b)**; ACE index **(c)**; Shannon index **(d)**; Simpson index **(e)**. Principal coordinate analysis plots of the urinary microbiota based on the weighted UniFrac **(f)**, unweighted UniFrac **(g)** and Bray-Curtis **(h)** distance metrics. Association of specific microbiota taxa with the diabetes group and control group was analyzed by LEfSe **(i)**. Genera enriched for controls in red and enriched for patients in blue. Only genera meeting a linear discriminant analysis score threshold > 2 are shown

All reads were classified into 6212 operational taxonomic units (OTUs). 3070 OTUs were identified in T2D patients and 3142 OTUs were identified in controls. Comparisons of alpha diversity between T2D patients and controls were shown in Table 1. There were no significant differences in Observed Species (88(11, 245) vs. 135(15, 258)), Chao 1 index (103.60(14.00, 272.00) vs. 149.50(29.00, 339.00)), ACE index 104.30(16.50, 416.70) vs. 146.10(40.70, 428.60), Shannon index (1.70(0.04, 3.48) vs. 1.78(0.01, 3.54)) and Simpson index (0.31(0.06, 0.99) vs. 0.38(0.07,1.00)) between diabetes group and control group (Table 1, Fig. 1a, e), which indicated that neither bacterial richness nor species diversity was not significantly changed in the T2D cohort.

As shown in Fig. 1f – h, we found urinary microbiota in diabetes group and control group mainly clustered together in the different area of the PCoA plots, respectively. Statistical differences were noted in weighted UniFrac (*P* = 0.045) as well as unweighted UniFrac (*P* = 0.010) distance metrics. However, the difference in Bray Curtis distance metrics could not reach statistical significance (Fig. 1h, *P* = 0.053).

We found that 10 genera were over-represented in the T2D cohort including *Escherichia-Shigella, Klebsiella, Aerococcus, Delftia, Enterococcus, Alistipes, Stenotrophomonas, Micrococcus, Deinococcus* and *Rubellimicrobium,* while 7 genera were under-represented including *Gallicola, Arcobacter, Arcanobacterium, Kocuria, Murdochiella, Solitalea* and *Peptoniphilus* (Fig. 1i). Among the genera mentioned above, Metastats algorithm shown the significantly altered in *Delftia, Enterococcus, Stenotrophomonas, Micrococcus, Deinococcus, Solitalea* and *Peptoniphilus* (Additional file 1: Table S1).

### Comparisons of bioinformatics between T2D patients with no to mild LUTS and those with moderate to severe LUTS

**Fig. 2:**
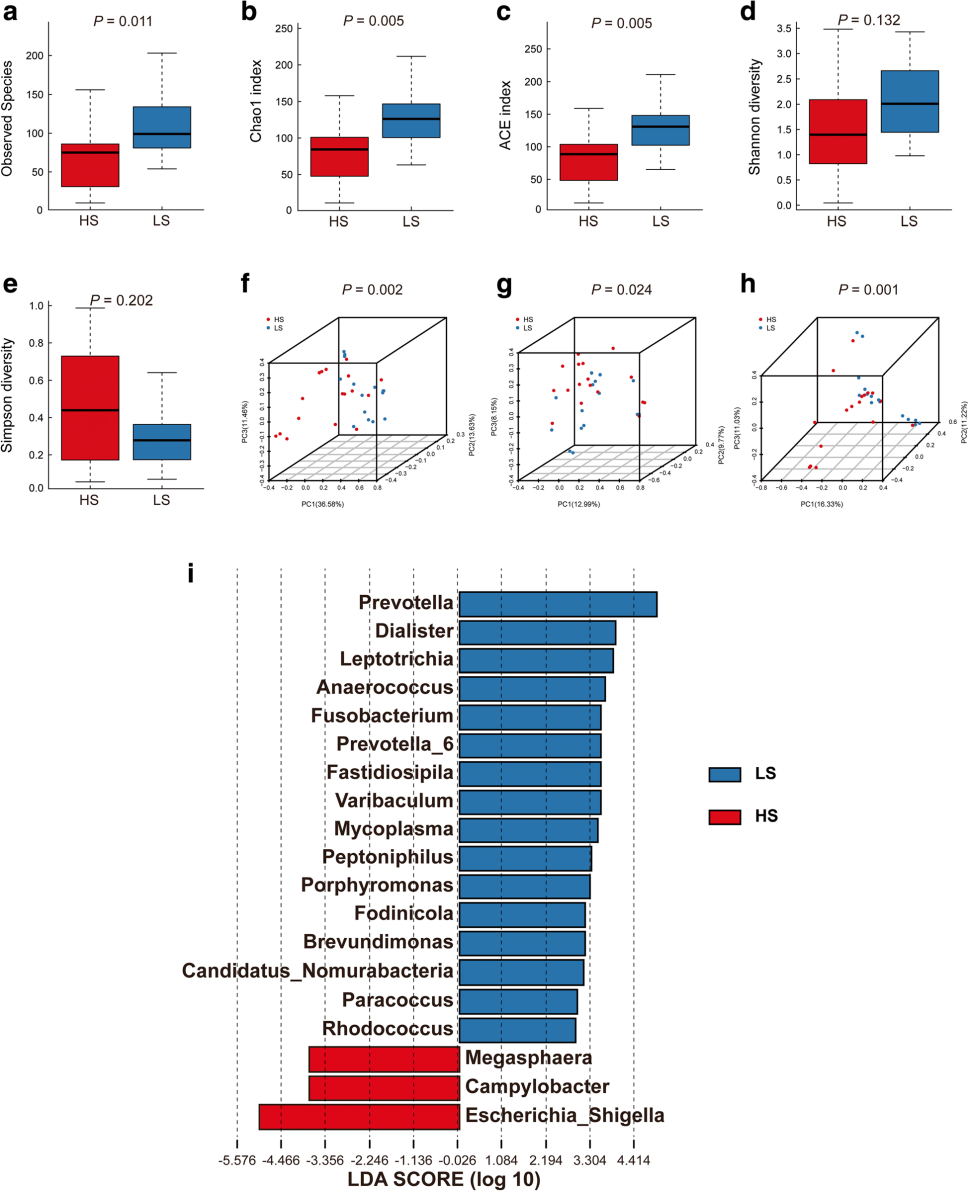
Alpha diversity and principal coordinate analysis for the HS group and LS group urinary microbiota. Observed Species **(a)**; Chao1 index **(b)**; ACE index **(c)**; Shannon index **(d)**; Simpson index **(e)**. Principal coordinate analysis plots of the urinary microbiota based on the weighted UniFrac **(f)**, unweighted UniFrac **(g)** and Bray-Curtis **(h)** distance metrics. Association of specific microbiota taxa with the HS group and LS group was analyzed by LEfSe **(i)**. Genera enriched for HS in red and enriched for LS in blue. Only genera meeting a linear discriminant analysis score threshold > 2 are shown

In order to detect the specific bacteria associated with the severity of LUTS, we next studied whether the urinary microbial profile was different between T2D patients with no to mild LUTS (LS group, total AUA-SI score ≤ 7) and T2D patients with moderate to severe LUTS (HS group, total AUA-SI score > 7). Higher Observed Species (Fig. 2a, 76(10, 175) vs. 98(54, 234), *P* = 0.011), Chao1 index (Fig. 2b, 83.86(11.00, 210.77) vs. 125.19(62.00, 251.77), *P* = 0.005), ACE index (Fig. 2c, 85.52(13.75, 204.84) vs. 129.82(63.89, 280.30), *P* = 0.005) were presented in the LS group, while no significant differences were observed in Shannon index (Fig. 2d, 1.40(0.04, 3.48) vs. 2.01(0.98, 3.43), *P* = 0.132) and Simpson index (Fig. 2e, 0.44(0.06, 0.99) vs. 0.28(0.07, 0.64), *P* = 0.202). This indicated that significantly decreasing bacterial richness was found in the HS group (Table 2). However, no correlation was observed between AUA-SI and alpha diversity.

**Table 2:** Comparisons of demographic and clinical characteristics as well as bacterial alpha diversity between HS group and LS group

	HS (*n* = 17)	LS (*n* = 15)	*P* value
Demographic Characteristics			
Age (y)	57.882 ± 8.230	55.933 ± 7.914	0.501
Body mass index (kg/m2)	23.715 ± 4.320	23.766 ± 4.596	0.975
Menstrual status [no.(%)]			1.000
Premenopausal	4 (23.5%)	4 (26.7%)	
Postmenopausal	13 (76.5%)	11 (73.3%)	
Reproductive status [no.(%)]			N/A
Fertile	17 (100%)	15 (100%)	
Sterile	0 (0%)	0 (0%)	
Clinical Characteristics			
Duration of diabetes (y)	6.882 ± 5.278	6.933 ± 4.250	0.976
Fasting blood glucose (mmol/L)	8.148 ± 3.798	7.647 ± 2.640	0.672
Hemoglobin A1c (%)	9.294 ± 2.044	6.720 ± 1.364	< 0.001
Retinopathy [no.(%)]	5 (29.4%)	5 (33.3%)	1.000
Peripheral neuropathy [no.(%)]	15 (88.2%)	12 (80.0%)	0.645
Hypertension [no.(%)]	7 (41.2%)	7 (46.7%)	0.755
Creatinine (μmol/L)	70.177 ± 68.789	59.467 ± 26.465	0.852
Estimated glomerular filtration rate (ml/min/1.73m^2^)	101.304 ± 34.613	108.150 ± 43.583	0.624
Parameter of Bacterial Alpha Diversity			
Observed species	76 (10, 175)	98 (54, 234)	0.011
Chao1	83.86 (11.00, 210.77)	125.19 (62.00, 251.77)	0.005
ACE index	85.52 (13.75, 204.84)	129.82 (63.89, 280.30)	0.005
Shannon index	1.40 (0.04, 3.48)	2.01 (0.98, 3.43)	0.132
Simpson index	0.44 (0.06, 0.99)	0.28 (0.07, 0.64)	0.202

Data were presented as mean ± SD for continuous variables and n (%) for counts in demographic and clinical characteristics. Parameters of bacterial alpha diversity were reported as median (range). HS, female type 2 diabetes patients with moderate to severe lower urinary tract symptoms. LS, female type 2 diabetes patients with no to mild lower urinary tract symptoms. N/A, not applicable. ※No data were missing

**Fig. 3:**
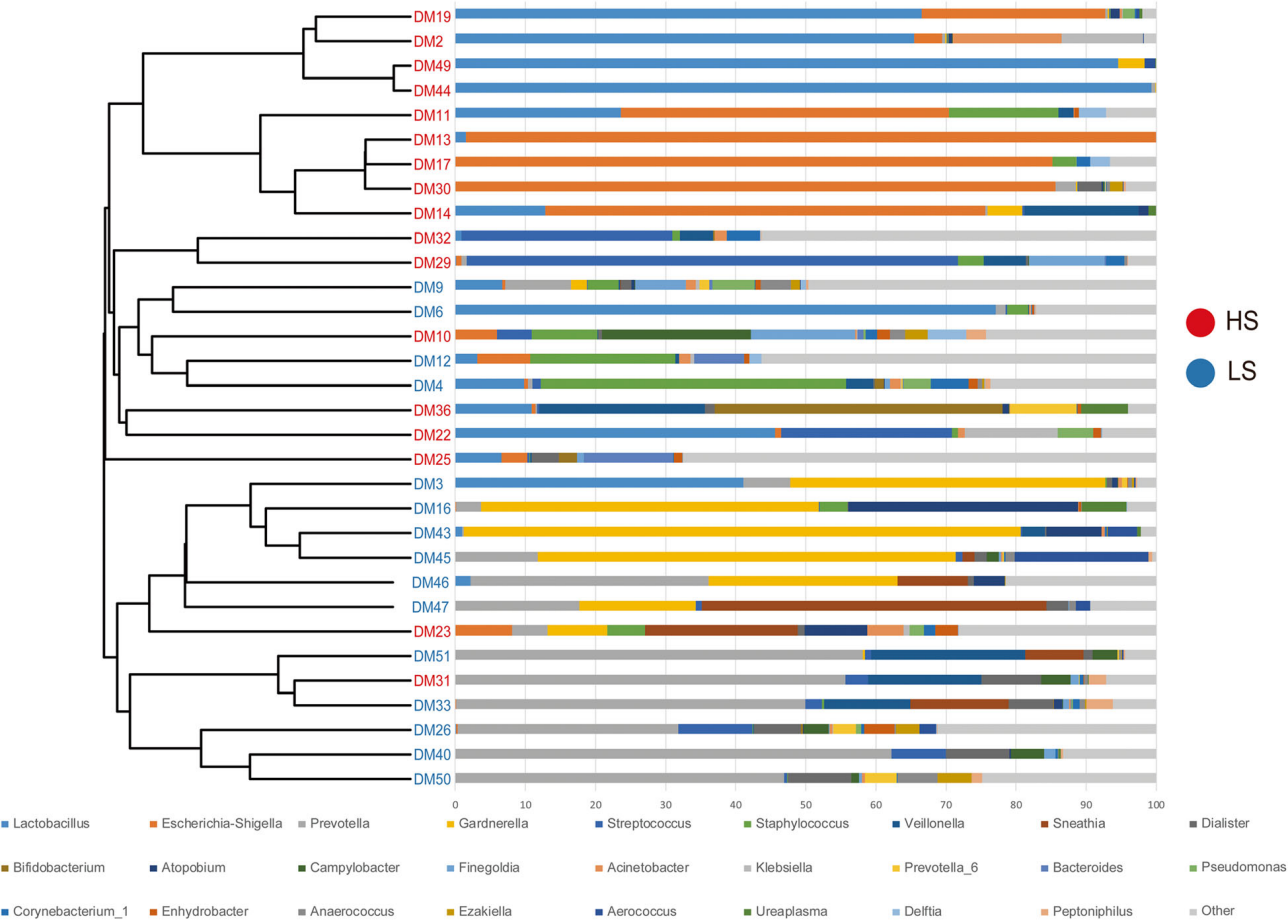
A clear hierarchical clustering of HS samples was observed in the dendrogram at the genus level (left; based on Bray Curtis distance metrics). In the histogram (right), each colored box represents a bacterial taxon and each bar, a subject. The height of a colored box represents the relative abundance of each organism in the sample. Bacterial genera with a relative abundance < 0.5% and unclassified genera are grouped as “Other”

PCoA plots demonstrated that samples from different groups were clustered together respectively and suggested that the urinary microbiota composition of LS group was distinct from that of HS group (Fig. 2f, h). Statistically differences were observed in MRPP test (*P* = 0.002, *P* = 0.024, *P* = 0.001 for weighted UniFrac, unweighted UniFrac and Bray Curtis distance metrics, respectively). In addition, a clear hierarchical clustering of LS samples and HS samples was observed on dendrogram which based on Bray Curtis distance metrics (Fig. 3).

LEfSe revealed that 3 genera were over-represented in the HS group including *Escherichia-Shigella, Campylobacter, Megasphaera*, while 16 genera were under-represented including *Prevotella, Dialister, Leptotrichia, Anaerococcus, Fusobacterium, Prevotella_6, Fastidiosipila, Varibaculum, Mycoplasma, Peptoniphilus, Porphyromonas, Fodinicola, Brevundimonas, Candidatus-Nomurabacteria, Paracoccus* and *Rhodococcus* (Fig. 2i). The Metastats analysis shown that *Escherichia-Shigella* was significantly more abundant in the HS group, while *Prevotella, Leptotrichia, Varibaculum, Mycoplasma,* and *Candidatus-Nomurabacteria* were more abundant in the LS group (Additional file 1: Table S1).

Of interest was that no significant differences were noted in clinical characteristics except Hemoglobin A1c (HbA1c) between the LS group and HS group (Table 2).

### Comparisons of bioinformatics between T2D patients with low HbA1c and high HBA1c

**Fig. 4:**
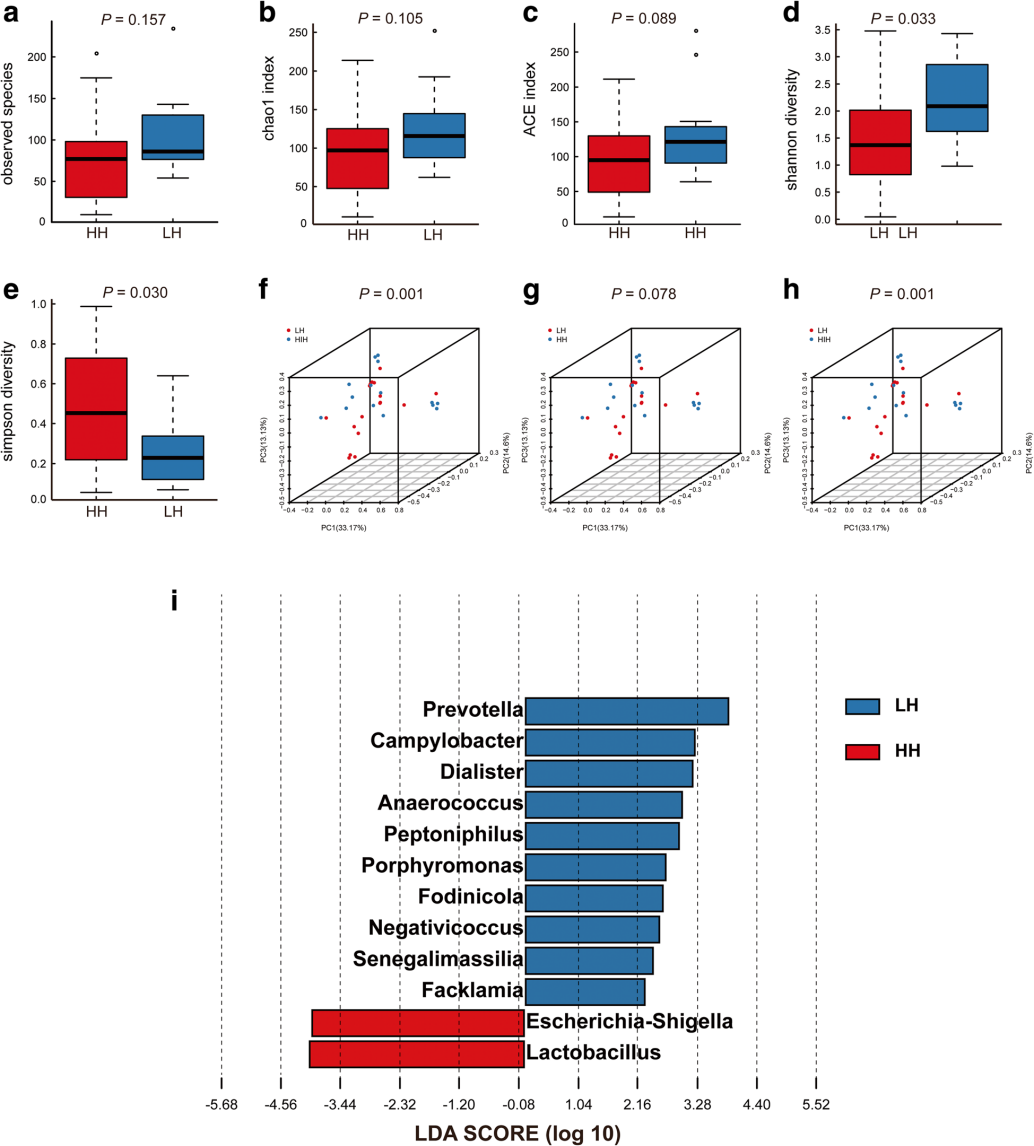
Alpha diversity and principal coordinate analysis for HH group and LH group urinary microbiota. Observed Species **(a)**; Chao1 index **(b)**; ACE index **(c)**; Shannon index **(d)**; Simpson index **(e)**. Principal coordinate analysis plots of the urinary microbiota based on the weighted UniFrac **(f)**, unweighted UniFrac **(g)** and Bray-Curtis **(h)** distance metrics. Association of specific microbiota taxa with HH group and LH group was analyzed by LEfSe **(i)**. Genera enriched for HH in red and enriched for LH in blue. Only genera meeting a linear discriminant analysis score threshold > 2 are shown

Due to the significant difference of HbA1c was found between LS group and HS group, we further analysis the characteristics of urinary microbiota between female T2D patients with low HbA1c (LH group, HbA1c ≤ 7%) and high HbA1c (HH group, HbA1c > 7%). Higher Shannon index (Fig. 4d, 1.369(0.044, 3.478) vs. 2.089(0.979, 3.429), *P* = 0.033) and lower Simpson index (Fig. 4e, 0.461(0.055, 0.987) vs. 0.229(0.069, 0.640), *P* = 0.029) were presented in LH group, while no significantly difference in Observed Species, Chao1 index and ACE index (Fig. 4a, 77(10,204) vs. 86(54,234), *P* = 0.157; Fig. 4b, 97.00(11.00, 213.55) vs. 115.67(63.89,245.84), *P* = 0.105; Fig. 4c, 94.87(0.26, 3.32) vs. 121.30(63.89, 280.30), *P* = 0.089). Comparison of alpha diversity indicated that significantly decreasing species diversity was discovered in HH cohort.

PCoA plots demonstrated that LH group samples and HH group samples were clustered respectively, which suggested that the urinary microbiota composition of LH group differed from that of HH group (Fig. 4f and Fig. 4h). MRPP test shown that the observed differences in PCoA plots were statistically significant (*P* = 0.001 for weighted UniFrac and Bray Curtis distance metrics, respectively). However, the difference in unweighted UniFrac distance metrics could not reach statistical significance (Fig. 4g, *P* = 0.078).

LEfSe demonstrated that 2 genera were over-represented in the HH group, including *Escherichia-Shigella* and *Lactobacillus*, while 10 genera were over-represented in the LH group, including *Prevotella, Campylobacter, Dialister, Anaerococcus, Peptoniphilus, Porphyromonas, Fodinicola, Negativicoccus* (Fig. 4i). Among these genera, Metastats algorithm shown the significantly different in *Anaerococcus, Fodinicola, Lactobacillus, Peptoniphilus, Senegalimassilia* (Additional file 1: Table S1).

**Fig. 5:**
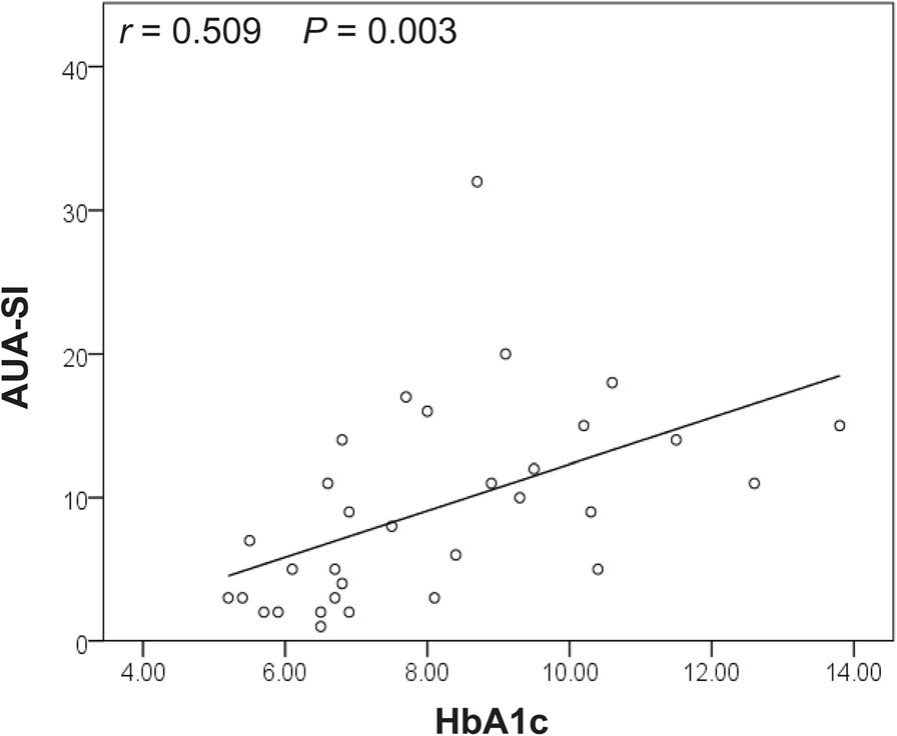
Correlations between AUA-SI and Hemoglobin A1c in female type 2 diabetes patients. Higher Hemoglobin A1c positively correlated with the AUA-SI score

Higher scores of AUA-SI (total score, 13.059 ± 6.805 vs. 4.867 ± 3.796; storage score, 7.824 ± 4.825 vs. 2.600 ± 2.230; emptying score, 5.235 ± 5.118 vs. 2.267 ± 3.369) were found in HH group (*P* < 0.05, Additional file 2: Table S2). Besides, HbA1c was found positively correlated with the total score of AUA-SI based on Pearson’s correlation (Fig. 5, *r* = 0.509, *P* = 0.003).

## Discussion

In this study, we describe the urinary microbiota in the female with and without T2D, female T2D patients with no to mild LUTS and moderate to severe LUTS as well as patients with low HbA1c and high HBA1c using 16S rDNA sequencing. Based on other reports [10, 11, 21] and our data, this is further confirmation that the commonly held clinical belief that healthy urine should be sterile is false.

Consented with Liu’s study, our results indicated that the composition of urinary microbiota may be impacted by diabetes [14]. (1) Samples from T2D patients and those from controls clustered separately in PCoA plots (Fig. 1f, h), which suggested a common urinary dysbiosis was associated to diabetes; (2) 10 genera were over-represented in T2D patients, including common pathogens such as *Escherichia-Shigella, Klebsiella* and *Enterococcus*; (3) it is found that female T2D patients suffering from much serious LUTS. Taken together, these results indicated that urinary dysbiosis (e.g. significantly increasing pathogens) may contribute to the initiation or development of cn-LUTS in type 2 diabetic patients. Common pathogens that over-represented in T2D patients would be discussed below.

Urinary microbial profile between LS and HS group was further analyzed. We found that the composition of urinary microbiota in the LS group was also significantly different from that in the HS group (Fig. 2f, 2i, Fig. 3). Hence, this raises the possibility that specific microbial patterns may be associated with more serious LUTS or the progression of LUTS in the female T2D patients. We may find out the specific urinary microbiota (Fig. 2i) that might exacerbate diabetes-induced cn-LUTS. The relatively increasing and decreasing genus would be also discussed in the following passage.

Due to the significant difference of HbA1c was found between the LS group and HS group, urinary microbial characteristics in the HH group and LH group were also analyzed. Statistical differences of urinary microbiota composition between HH and LH cohort were found in our analysis (Fig. 4f, i). In Liu et al.’s study, they found that increased *Actinobacteria* phylum as well as decreasing *Akkermansia muciniphila* were positively correlated with fasting blood glucose [14]. It is therefore suggested that poorly controlled blood glucose might further influence the composition of urinary microbiota. Furthermore, we found that most patients in the HH group suffered from more serious LUTS and HbA1c was positively correlated with AUA-SI. These raise the possibility that poorly controlled blood glucose might exacerbate cn-LUTS. As a result, we suggested the intriguing hypothesis that chronic hyperglycemia might induce or deteriorate cn-LUTS in T2D patients through influencing the urinary microbiota composition. Although the cause-effect relationship among hyperglycemia, urinary microbiota and cn-LUTS cannot be determined under our study, it is worth further researching.

In both cohorts, one or two genera dominated most of the sequence profiles. *Escherichia-Shigella* was relatively increased in diabetes group and HS group. *Klebsiella* and *Enterococcus* were relatively increased in the diabetes group. *Klebsiella* is a common conditional pathogenic bacterium that causing respiratory and urinary tract infections when host immunity is impaired [22]. *Enterococcus* is a nosocomial pathogen which can cause UTI and endocarditis [23]. Increased TNF-α, a proinflammatory cytokine, was discovered in the bladder tissue of T2D mice [24]. Toll-like receptors 4 pathway was also activated in type 1 diabetes rats induced by Streptozotocin [25]. It was a strong indication that inflammation existed in the bladder tissue of diabetic animals. In the present study, diabetic patients that included were suffered from cn-LUTS. As for these patients, standard clinical cultivation procedures have a too low sensitivity to detect the uropathogens. 16S rDNA sequencing of mid-stream urine samples might represent a novel method for diagnosing “UTI” (urinary dysbiosis).

The trends of *Prevotella* abundance changes were variable in different researches. In our study, *Prevotella* was decreasing in HS cohort and our previous study shown that decreasing *Prevotella* was found in overactive bladder patients [10]. However, Pearce et al. revealed that enrichment of *Prevotella* was observed in urgency urinary incontinence patients [11]. 16S rDNA sequencing-based approach can’t identify bacteria well at the species level and over 20 species have been already classified into *Prevotella*. It is hypothesized that various species play distinct roles in different disorders, leading to the contradiction mentioned before. Thus, it may be necessary to explore the role of *Prevotella* in urologic disorders via whole genome shotgun sequencing or expanded urine culture.

Enrichment of *Lactobacillus* was presented in the HH group. *Lactobacillus* is considered being low virulence, hardly causing infections in human beings [12]. It is best known for its dominance in vaginal microbiota and it can prevent vaginitis by maintaining a physiological acidic environment in the vagina. Because of these properties, *Lactobacillus* has been applied to prevent or even treat UTI [26]. However, there is increasing evidence that specific *Lactobacillus spp* may be pathogenic and linked to UTI [27, 28]. It has been suggested that *Lactobacillus* is resistant to the widely used antibiotics, which can reproduce during the treatment of UTI and invade the bladder to induce inflammation [29]. In addition, several studies found that *Lactobacillus* was closely associated with cn-LUTS [27, 28]. For example, Maskell et al. found that *Lactobacillus* gradually disappeared (detected by special culture techniques) as symptoms subsided after the use of antibiotics for treating LUTS in the female patients who had a negative urine culture before [30]. The result suggested that a shift in the microbial community towards *Lactobacillus* in the HH group may be an important etiological factor for the more serious LUTS reported by the patients.

In this study, no significant differences in alpha diversity were found between T2D patients and controls but decreased richness as well as species diversity were shown in HS and HH group, respectively. Although a reduction in alpha diversity has been thought as a feature of gastrointestinal diseases, such as ulcerative colitis, Crohn’s disease and colorectal cancer [31–33] as well as an increased vaginal microbiota diversity was associated with bacterial vaginitis [34]. No consistent changes in alpha diversity were found in urologic disorders. Increased alpha diversity was observed in urgency urinary incontinence [11], decreasing alpha diversity was demonstrated in overactive bladder [10], while no significant difference was noted in prostate cancer [35]. In Liu et al.’s study, they reported that urinary microbiota diversity and richness were lower in female T2D patients [14]. Demographic features, exclusive criteria and less microbial species in the urinary tract may be the reasons leading to this contradiction between Liu’s study and ours [14].

Owing to the urine samples may be contaminated with microbiota surrounding the urethral orifice, mid-stream urine was collected by the clean catch method with labial separation supervised by the author (Weina Huang). Catheter-derived urine samples were suggested as an alternative method, but the method was considered being invasive and was not ethically workable in patients who do not have a clinical indication for it. Comparison of results from Siddiqui et al.’s urinary microbiota study on female mid-stream urine with the consequences of suprapubic aspirate by Wolfe et al. shown that the major findings were the same [12]. It was a strong indication that results from mid-stream urinary microbiota were reliable.

Participants who used antibiotics recently have been excluded, however, numerous drugs may influence gut microbiota composition, and their effect may be present even in the urinary microbiota. Anti-diabetic drugs, particularly metformin, also have a well-known effect on the gut microbiota composition [36]. Although all the T2D patients included in our study were hospitalized and most of them were treated with insulin (data not shown), we could not fully avoid the interference of anti-diabetic drugs. The correlation between urinary microbiota and anti-diabetic drugs should be studied.

Our study is not devoid of limitations. Firstly, it is impossible to determine the cause-effect relationship between symptoms and bioinformatics indicators in this cross-sectional study. Thus, prospective studies with a larger sample and animal experimentation will be needed to clarify the role of urinary microbiota in the development and progression of cn-LUTS in female T2D patients. Secondly, we cannot confirm that the urinary microbes characterized by 16S rDNA sequencing were actually viable. We should add expanded urine culture as a complementary method and make a comprehensive analysis of information that provided by expanded urine culture and 16S sequencing in the future. Finally, the heterogeneity of our cohort should not be ignored because of wide the range of age and duration of diabetes in our cohort. Thus, patients might be stratified by age and time of symptoms in a larger cohort.

## Conclusions

We have comprehensively profiled the urinary microbiota associated with cn-LUTS in female T2D patients. The link between urinary microbiota and cn-LUTS in female T2D patients without evidence of UTI has been established but the cause-effect relationship is still unclear. A better understanding of urinary microbiota in the development and progression of cn-LUTS in female T2D patients was necessary, which might provide novel diagnostic biomarkers as well as microbiota-targeted therapeutic options. The severity of cn-LUTS might be related to hyperglycemia and chronic hyperglycemia might induce or promote cn-LUTS by influencing the composition of urinary microbiota.

## Additional files

Additional file 1: **Table S1.** Differences of urinary microbiota between groups at genus level based on Metastats algorithm (XLSX 12 kb)

Additional file 2: **Table S2.** Comparisons of demographic and clinical characteristics between HH group and LH group (DOCX 16 kb)

## Data Availability

The datasets used and/or analyzed during the current study are available from the corresponding author on reasonable request.

## Abbreviations

AUA-SI: American Urological Association Symptom Index questionnaire
cn-LUTS: Cultured negative lower urinary tract symptoms
HbA1c: Hemoglobin A1c
LEfSe: Linear discriminant analysis effect size
LUTS: Lower urinary tract symptoms
MRPP: Multiple Response Permutation Procedure
OTUs: Operational taxonomic units
PCoA: Principal coordinate analysis
QIIME: Quantitative Insights Into Microbial Ecology
T2D: Type 2 diabetes
UTI: Urinary tract infections
