# Supplementary material for: Relationship between alterations of urinary microbiota and cultured negative lower urinary tract symptoms in female type 2 diabetes patients
Source: BMC Urol. 2019 Aug 22;19:78. doi: 10.1186/s12894-019-0506-0 (PMC6704724; doi:10.1186/s12894-019-0506-0)
Supplement: Supplementary file 2 — Table S2. Comparisons of demographic and clinical characteristics between HH group and LH group (DOCX 16 kb) [file 12894_2019_506_MOESM2_ESM.docx]

| **Table S2. Comparisons of demographic and clinical characteristics between HH group and LH group** | | | |
| --- | --- | --- | --- |
|  | HH  (n = 17) | LH  (n = 15) | *P* value |
| **Demographic Characteristics** |  |  |  |
| Age (y) | 57.941±8.227 | 55.867±7.900 | 0.474 |
| Body mass index (kg/m2) | 23.462±3.529 | 24.053±5.294 | 0.710 |
| Menstrual status [no.(%)] |  |  | 0.692 |
| Premenopausal | 5 | 3 |  |
| Postmenopausal | 12 | 12 |  |
| Reproductive status [no.(%)] |  |  |  |
| Fertile | 17(100%) | 15(100%) | N/A |
| Sterile | 0(0%) | 0(0%) |  |
| **Clinical Characteristics** |  |  |  |
| Duration of diabetes (y) | 7.059±4.981 | 6.733±4.636 | 0.850 |
| Fasting blood glucose (mmol/L) | 7.716±2.694 | 8.137±3.899 | 0.722 |
| Retinopathy [no.(%)] | 4 | 6 | 0.450 |
| Peripheral neuropathy [no.(%)] | 13 | 14 | 0.338 |
| Hypertension [no.(%)] | 7 | 7 | 0.755 |
| Creatinine (μmol/L) | 69.647±68.607 | 60.067±27.241 | 0.852 |
| Estimated glomerular filtration rate (ml/min/1.73m^2^) | 100.918±32.014 | 108.587±45.723 | 0.583 |
| American Urological Association Index |  |  |  |
| Total score | 13.059±6.805 | 4.867±3.796 | <0.001 |
| Storage score | 7.824±4.825 | 2.600±2.230 | <0.001 |
| Emptying score | 5.235±5.118 | 2.267±3.369 | 0.002 |

Data were presented as mean ± SD for continuous variables and n (%) for counts in demographic and clinical characteristics. HH, female type 2 diabetes patients with high Hemoglobin A1c (Hemoglobin A1c > 7%). LH, female type 2 diabetes patients with low Hemoglobin A1c (Hemoglobin A1c ≤ 7%). N/A, not applicable. ※No data were missing.
